# Real-Time Tool Localization for Laparoscopic Surgery Using Convolutional Neural Network

**DOI:** 10.3390/s24134191

**Published:** 2024-06-27

**Authors:** Diego Benavides, Ana Cisnal, Carlos Fontúrbel, Eusebio de la Fuente, Juan Carlos Fraile

**Affiliations:** Instituto de las Tecnologías Avanzadas de la Producción (ITAP), Escuela de Ingenierías Industriales, Universidad de Valladolid, Paseo Prado de la Magdalena 3-5, 47011 Valladolid, Spain; diego.benavides@estudiantes.uva.es (D.B.); carlos.fonturbel@uva.es (C.F.); efuente@uva.es (E.d.l.F.); jcfraile@uva.es (J.C.F.)

**Keywords:** artificial intelligence, biomedical image processing, laparoscopy robotic surgery, real-time, convolutional neural network, surgical tool tracking

## Abstract

Partially automated robotic systems, such as camera holders, represent a pivotal step towards enhancing efficiency and precision in surgical procedures. Therefore, this paper introduces an approach for real-time tool localization in laparoscopy surgery using convolutional neural networks. The proposed model, based on two Hourglass modules in series, can localize up to two surgical tools simultaneously. This study utilized three datasets: the ITAP dataset, alongside two publicly available datasets, namely Atlas Dione and EndoVis Challenge. Three variations of the Hourglass-based models were proposed, with the best model achieving high accuracy (92.86%) and frame rates (27.64 FPS), suitable for integration into robotic systems. An evaluation on an independent test set yielded slightly lower accuracy, indicating limited generalizability. The model was further analyzed using the Grad-CAM technique to gain insights into its functionality. Overall, this work presents a promising solution for automating aspects of laparoscopic surgery, potentially enhancing surgical efficiency by reducing the need for manual endoscope manipulation.

## 1. Introduction

Surgical robotics has emerged as a transformative force in modern medicine, revolutionizing the way complex procedures are performed. Its significance lies in its capacity to enhance surgical precision, minimize invasiveness, and improve patient outcomes. Robotic laparoscopic surgery has garnered widespread acceptance over the years due to its numerous benefits, including reduced post-operative pain, shorter hospital stays, and faster recovery times [1]. However, the prohibitively high cost of robots capable of performing complete surgeries makes them unaffordable for many medical centers [2].

Robotic assistant systems, inspired by the co-worker concept [3], offer cost-effective alternatives to fully teleoperated systems like the da Vinci. Examples include Stryker’s Mako (Kalamazoo, MI, USA) [4] and Robodoc (Curexo, Seoul, Republic of Korea) [5] for joint replacement procedures and Rosa (Zimmer Biomet, Zug, Switzerland) [6] for neurological and spine surgeries. The development of a robotized camera holder for laparoscopy has been a widely discussed topic since the 20th century, with the introduction of several functional devices [7,8]. Another more recent example is the ViKY (EndoControl, La Tronche, France), which is manually positioned at the trocar and secured via a poly-articulated support [9]. Additionally, a seven-DoF commercial robot was proposed as a camera holder, utilizing the gaze gestures of the surgeon to control camera movements [10]. However, these solutions require the surgeon to continually provide motion instructions to the robot assistant, falling short of meeting the requirements of the surgical community.

In recent years, the detection and tracking of surgical instruments based on image analysis have gained prominence. This approach utilizes endoscopic images to estimate the tool’s position in a simple and flexible manner, without the need for additional equipment, workflow changes, or motion instruction from the surgeon. Within this context, various methods have been proposed, with a notable shift towards deep learning-based detection methods due to the rapid advancement of artificial intelligence.

In fact, object detection methods have been the subject of an enormous amount of research due to their practical application in all areas, from autonomous vehicle guidance to surgical tool tracking in operations. Traditional techniques experienced a significant breakthrough with the advent of convolutional neural networks (CNNs) a decade ago. Girshick et al. [11] were the first to apply this approach using a region-based convolutional neural network (R-CNN). Three years later, the presentation of Faster R-CNN [12] marked a significant improvement in execution time compared to existing algorithms. Faster R-CNN introduced a distinct network known as the Region Proposal Network to suggest potential regions containing objects of interest, which are later fed into the classification network. Additionally, Faster R-CNN introduced the integration of nine anchor boxes per pixel in the feature map to accommodate variations in scale and aspect ratio. Faster R-CNN has been employed to achieve surgical tool localization and evaluate surgeons’ techniques [13,14].

Faster R-CNN approaches object detection as a two-stage problem: generating region proposals and then classifying those regions. However, more recent algorithms consider object detection from an integrated perspective by introducing the input image into a single convolutional network that predicts bounding boxes and their probabilities in a single stage. The You Only Look Once (YOLO) algorithm [15] implements this unified approach by framing object detection as a regression problem. YOLO is recognized as one of the most efficient algorithms, suitable for real-time processing, due to its single convolutional network evaluation. The YOLO architecture was introduced by Choi et al. [16] for real-time surgical instrument tracking. They achieved 72.26% mean average precision on a dataset that included seven surgical tools. The convolutional layers were pretrained using the ImageNet 1000-class competition dataset, and then a gallbladder surgery image dataset was used for the learning process. Choi et al. finally concluded that the low precision in the detection of some specific instruments was due to the insufficient number of images to learn from.

Although single-stage detectors such as YOLO show more efficiency than their two-stage counterparts, both approaches rely on anchor boxes. These anchor boxes introduce numerous hyperparameters that require fine-tuning, hindering the network training process. Despite their high accuracy in surgical tool detection, methods utilizing anchor boxes often fall short in real-time applications. To address this limitation, Liu et al. [17] combined an anchor-box-free CNN with an Hourglass network [18], facilitating real-time surgical tool localization through heatmap estimation. Models based on U-Net architectures [19], widely popular in segmentation tasks, have also been used to determine the position of surgical instruments [20,21]. Kurmann et al. proposed a U-shaped network to simultaneously recognize multiple instruments and their parts [22]. Laina et al. [23] formulated the position estimation task as heatmap regression, estimated concurrently with tool segmentation.

This article presents a vision model for real-time tool localization, employing two Hourglass modules in series. Building on our previous work [24], which introduced a novel force-based control strategy utilizing pivoting motions instead of the regular remote center of the tool (RCM) for the camera holder, this paper aims to advance this research. Our ultimate goal is to develop a fully autonomous robotic camera holder that integrates this mobility approach with the proposed vision model. To this end, this paper focuses on the design, development, and evaluation of the deep learning model with a focus on providing real-time tool localization for a surgical robotic system.

The paper is structured as follows: Firstly, it details the databases utilized, the architecture of the model employed, the performance metrics used for evaluation, and the visual-based Grad-CAM method used for model interpretability. Subsequently, it presents the model performance and an in-depth analysis employing the Grad-CAM method. Furthermore, the implications of the findings are discussed, along with potential avenues for future research, with a particular focus on improving model accuracy and robustness in real-world surgical scenarios. Finally, the key findings of this study are summarized, reiterating the significance of the proposed vision model for advancing robotic-assisted laparoscopic surgery.

## 2. Materials and Methods

**Figure 1:**
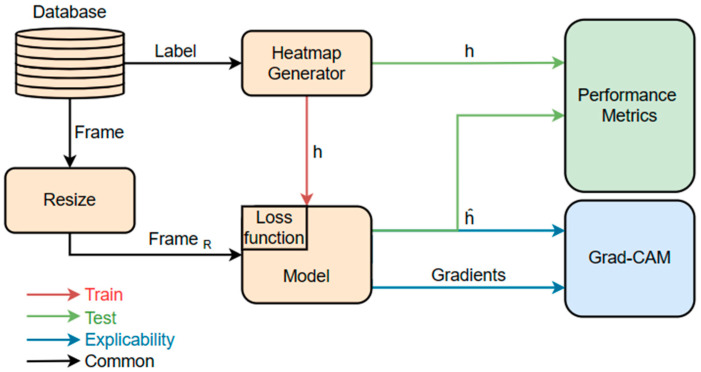
Diagram of the tool localization model development process.

For the development and validation of the tool localization model, three databases were utilized. Frames extracted from these databases were resized before being fed into the proposed model. Additionally, for training purposes, labels in the form of heatmaps were generated and introduced into the model. To evaluate the model, a series of evaluation metrics were employed to compare the predicted heatmap $\left(\hat{h}\right)$ and the ground truth heatmap (h). The schematic representation of this process is depicted in Figure 1. This section details (1) the databases, (2) the developed model (architecture and loss function) and image pre-processing, encompassing rescaling and heatmap generation for labeling, (3) the performance metrics, (4) the Grad-CAM method employed for the visual explanation of the model.

### 2.1. Databases

In this study, three databases containing up to two rigid surgical instruments were employed to train and evaluate the proposed tool detection algorithm:

1. ITAP Medical Robotics dataset

**Figure 2:**
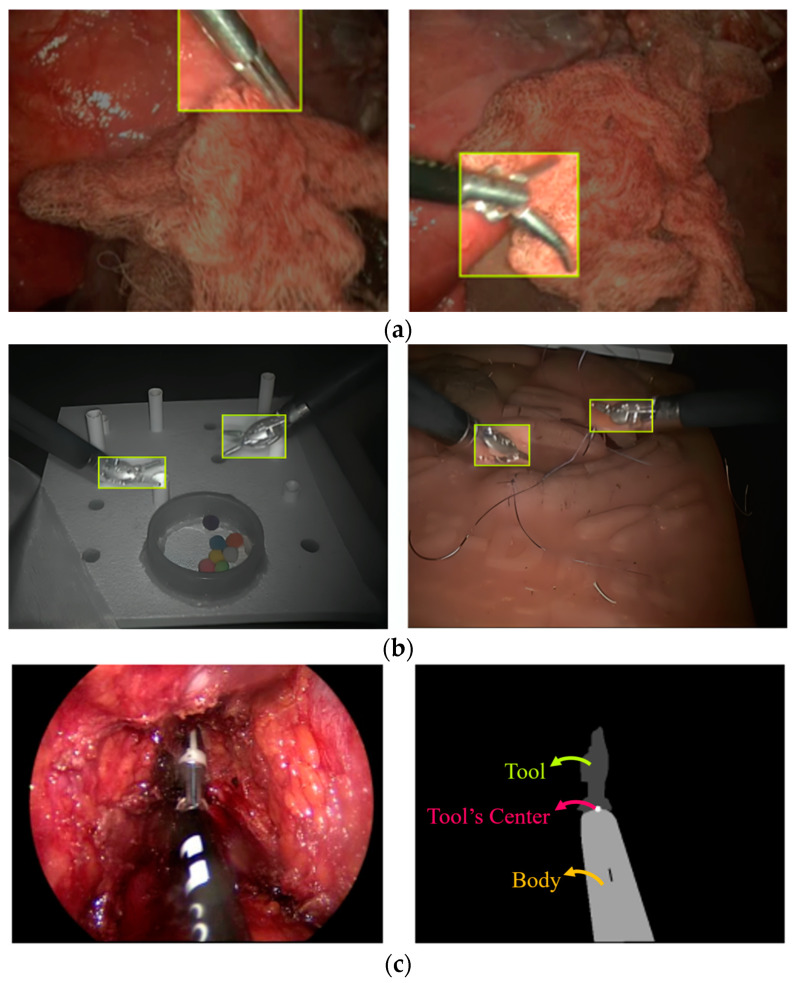
Sample images from (**a**) ITAP Medical Robotics Group (simulated surgical scenes with porcine ex vivo organs), (**b**) ATLAS Dione (operational tasks with simulators and objects), and (**c**) EndoVis Challenge (ex vivo surgical simulators) datasets, with corresponding annotations: bounding boxes (**a**,**b**) and tool center coordinates (**c**).

The ITAP dataset [25] includes 3532 frames extracted from simulated surgical scene videos (Figure 2a). The simulated surgical procedures involved the manipulation of various porcine organs ex vivo, employing the surgical tool Clickline (Karl Storz, Tuttlingen, Germany). Among the recorded 3532 frames, only 609 frames contain the surgical tool based on frame labels. The videos were captured using a Storz Telecam One-Chip Camera Head in conjunction with the HOPKINS telescope 0° (Karl Storz, Germany). Each frame exhibits a resolution of 720 × 576 pixels, and labels are represented as bounding boxes.

2. ATLAS Dione dataset

The ATLAS Dione dataset [13] comprises 99 study videos wherein ten surgeons execute six different operational tasks employing the da Vinci Surgical System (Figure 2b). Each frame maintains a resolution of 854 × 480 pixels and is accompanied by annotations for surgical tools, including tool type and the positional coordinates of bounding box vertices. While the incorporation of manikin simulators and objects for movement simulations may enhance the generalizability of the model, it introduces a notable limitation by deviating from real-world scenarios.

3. EndoVis Challenge dataset

The EndoVis’15 dataset [26] comprises 4535 frames, out of which 180 are annotated. These images correspond to four ex vivo surgical simulations (Figure 2c). The labels for these images are provided by coordinates of the tool center, which is located between the rigid part and the tool of the surgical instrument.

An overview of the characteristics of the three datasets is provided in Table 1. While the ITAP and EndoVis Challenge datasets provide the most realistic images of ex vivo surgical procedures, their primary drawback lies in the low number of labeled images featuring tool presence: 609 for the ITAP dataset and 180 for the EndoVis Challenge dataset. This makes it unlikely to effectively train a model, as a large number of images are required. In contrast, the ATLAS Dione dataset contains a significant number of labeled images (22,467), albeit with the significant disadvantage of non-realistic images. Furthermore, there is a notable disparity in the type of label provided by the datasets. While the ITAP and ATLAS Dione datasets offer a bounding box encompassing the tool, typically positioning it approximately at the center of the distal portion of the surgical instrument, the EndoVis Challenge dataset considers the center of the tool as the boundary between the rigid part of the surgical instrument and the tool itself, located in the most distal area. This difference in interpreting the center of the tool in the labeled data could pose challenges in training the model and comparing performance.

### 2.2. Tool Localization Model

#### 2.2.1. Network Architecture

**Figure 3:**
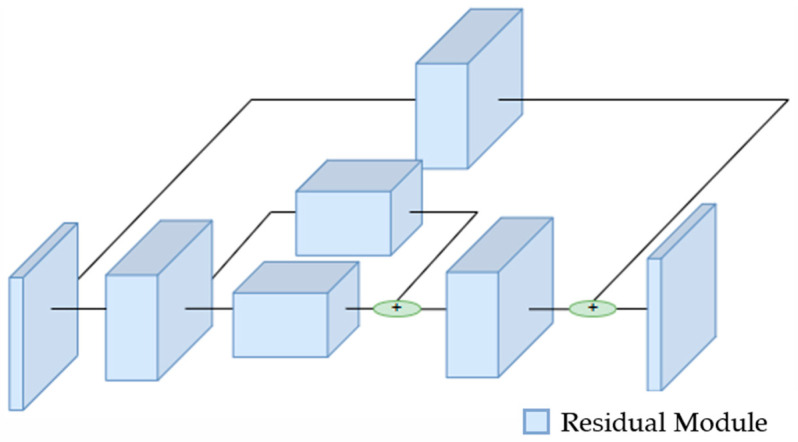
Basic structure of an Hourglass module. Each block corresponds to a residual module in the original model.

The proposed model is founded upon the Hourglass network, a CNN widely employed for tasks involving the localization of key points in images. This architectural framework was originally introduced by Newell et al. [18] to address the challenge of preserving intricate information across diverse spatial scales within a deep neural network using several Hourglass modules (Figure 3). Specifically, the proposed network is based on two individual Hourglass modules arranged in series. Each individual module consists of a down-sampling and an up-sampling stage for feature map dimensionality reduction and expansion, akin to the U-Net model or autoencoder structures. Unlike the U-Net model, this architecture incorporates skip connections with an intermediate processing stage. The main difference of this module from other traditional architectures lies primarily in its greater symmetry between the stages of feature map dimensionality reduction and expansion. Additionally, typical operations in common up-sampling stages in other architectures, such as transposed convolution, are replaced in this module by an up-sampling layer using the k-means algorithm, resulting in lower computational cost as these layers are non-trainable.

The basic structure of the model consists of a 7 × 7 convolution module and a residual module, which together reduce the dimensionality of images by a factor of 4. Subsequently, the images are fed into each of the two Hourglass modules, reducing their dimensionality before increasing it again as explained earlier. Next, the output is fed into a simple forward-propagating CNN to interpret the features extracted by each Hourglass module. After the extraction of features from the first Hourglass module, the previous feature maps are brought back to the output for combination with the extracted features. This is accomplished using a 1 × 1 convolution and a batch-normalization layer for supervision. Finally, the maps re-enter a second Hourglass module identical to the first, and inference is performed with a CNN identical to that explained in the first module. In this case, the outputs of the three branches of the CNN are not summed; each output refers to a specific feature: tool center, bounding box location, and offset. Although the model is designed to capture these three features, given that the sole objective is to locate the tool center, only the branch relevant to the tool center has been preserved to optimize parameter usage and temporal performance.

Additionally, the residual modules found in the original Hourglass architecture are replaced by fire modules. These modules exhibit slight variations from residual modules, particularly in terms of computational cost. While residual modules perform 3 × 3 convolutions to capture both spatial and channel-wise relationships, fire modules split these operations to prioritize channel-wise relationships before spatial relationships, thus enhancing efficiency. This is achieved through a sequence where the number of channels is first reduced via a 1 × 1 convolution, followed by a bifurcation involving parallel execution of a 1 × 1 convolution akin to residual modules and a separable convolution. Such an approach proves more efficient compared to traditional convolutions, as it assigns a dedicated set of filters to each channel, eliminating the need for linear combinations across channels [27].

Finally, the model generates two monochromatic images, each measuring 128 × 128 pixels and possessing a single intensity channel. Employing a sigmoid function as the activation function in the final layer, the model assigns a probability to each pixel, indicating its likelihood of representing the tool center. Consequently, the output images serve as heatmaps delineating the spatial distribution of tool centers, akin to a two-dimensional Gaussian function, with the highest intensity pixel corresponding to the tool center.

#### 2.2.2. Image Pre-Processing

1. Input image resize

Prior to the image processing within the neural network, the input image must be resized to match the model’s input size (height, width, channels). Note that the third dimension of channels corresponds to three channels, representing the RGB channels. Additionally, the images were normalized to standardize the intensity levels across images, thereby preventing variations in image intensity from affecting the performance of the model.

2. Heatmap generation

**Figure 4:**
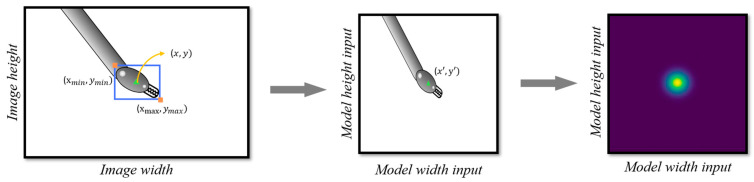
Image pre-processing involving image resizing followed by heatmap generation.

The objective of the network is to produce heatmaps wherein maximum values correspond to tool centers. Hence, it is imperative to generate heatmaps for training purposes, which also implies image resizing (Figure 4). First, the label coordinates, originally in pixels relative to the original image size, are resized to match the network’s output layer size (128 × 128 px). This process involves applying a rescale factor to each axis based on the original image size, ultimately determining the coordinates of the tool’s center within the new image dimensions.

The model can detect up to two tools (*N* = 2), requiring the generation of two heatmaps, one for each tool. In scenarios where only one tool is present (*N* = 1), one heatmap will contain nonzero values, while the other remains zero. In the absence of any tools (*N* = 0), both heatmaps will consist entirely of zeros. The heatmap generation process entails initializing a 128 × 128 grid with zero values. Subsequently, pixels corresponding to the center coordinates of the tool are assigned a value of one. Further refinement of the heatmap is achieved by applying a Gaussian filter, described by

(1) $$g\left(x,y\right)=\frac{1}{2\pi \sigma^{2}}e^{-\frac{\left(x^{2}+y^{2}\right)}{2\sigma^{2}}}.$$

The parameters of the Gaussian filter are tailored to the characteristics of the tool, with the standard deviation (*σ*) determined as a function of the tool’s dimensions. Specifically, the standard deviation is calculated using

(2) $$\sigma =\frac{\max(tool_{H},\;tool_{W})}{3}.$$

This methodology was previously employed by Hei Law et al. [28]; here, $tool_{H}$ is the tool height and $tool_{W}$ is the tool width.

#### 2.2.3. Loss Function

The model output is two heatmaps wherein pixels with maximum values serve as indicators of the centers of the tools. This task resembles a classification problem seen in image segmentation models, wherein each pixel is assigned a probability of representing a specific class. However, unlike typical segmentation tasks, the distribution of positive prediction points in this scenario is sparse, with only pixels in close proximity to the actual tool centers expected to have nonzero values. This sparsity poses a challenge, as there is a risk of the neural network converging towards predicting most pixels as zeros, resulting in high overall accuracy but failing to capture subtle variations in tool placement accurately. To address this issue, Lin et al. [29] proposed the focal loss function as a solution to handle the class imbalance commonly found in object detection datasets.

The balanced cross-entropy function weights the importance of positive/negative examples; however, it cannot differentiate between easy/hard examples. Therefore, a modulating factor ${\left(1-p_{t}\right)}^{\gamma }$ a is added to the cross-entropy loss function so the training focuses on hard classification examples and down-weights easy ones. The focal loss (FL) function is

(3) $$FL\left(p_{t}\right)=-\alpha_{t}{\left(1-p_{t}\right)}^{\gamma }\log(p_{t}),$$

where *p_t_* refers to the estimated probability for the positive class and Υ ≥0 is the tunable focusing parameter.

Law et al. [28] introduced a refined version of the focal loss function (FL′), incorporating an additional modulation factor depending on the actual pixel value. This adjustment mitigates the penalty imposed by the function in the vicinity of the tool’s center point, offering particular utility when dealing with labels generated by Gaussian filters. In such cases, where a single point possesses a unit value while the remaining points are close to one, misclassification would incur substantial penalization. This equation is defined as

(4) $$FL\prime (p_{t})=\frac{-1}{N}\sum_{c=1}^{C}\sum_{i=1}^{H}\sum_{j=1}^{W}\left\{\begin{array}{c} {\left(1-p_{cij}\right)}^{\alpha }\log\left(p_{cij}\right)\;\;\;\;\;if\;y_{cij}=1\\ {\left(1-y_{cij}\right)}^{\beta }{\left(p_{cij}\right)}^{\alpha }\log\left(1-p_{cij}\right)\;otherwise \end{array}\right.,$$

where *p_cij_* refers to the prediction of the heatmap *c* in the pixels (*i*, *j*). *y_cij_* refers to the ground truth heatmap *c* in the pixels (*i*, *j*). *α* is a positive value whose function is to modulate the class imbalance between positive (*y_cij_* = 1) and negative pixels (*y_cij_* ≠ 1). *β* is a positive value that has the purpose of decreasing the loss function value in pixels near the positive one (*y_cij_* = 1). In this case, a value of 2 has been used for *α* and 4 in the case of *β*.

### 2.3. Performance Metrics

The performance of the proposed model was assessed using three metrics: mean error, accuracy, and peak activation value (PAV). The first two metrics are based on the distance between the model’s prediction of the tool center and the actual center, while the third is related to the model’s confidence.

1. Mean error

**Figure 5:**
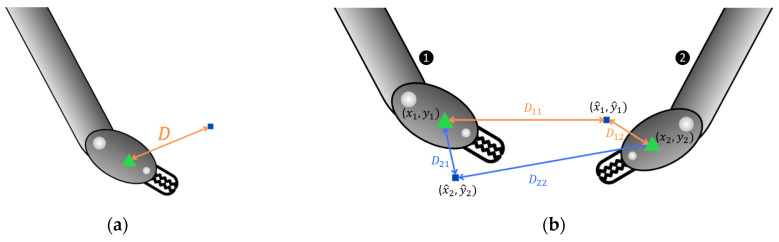
Representation of the Euclidian distance for scenarios with (**a**) one tool present and (**b**) two tools present. The green triangle represents the actual location, and the blue square represents the predicted location.

Let $h_{l}$ (where *l* = 1, 2) represent the ground truth heatmap, with $h_{l}(i,j)$ denoting the activation at position (i, j). The actual center of tool *l* is at coordinates $p_{l}$ (x_l_, y_l_), corresponding to the position of the maximum activation of $h_{l}$. Now, let ${\hat{h}}_{k}$ (where *k* = 1, 2) be the matrix representing the predicted heatmap, where ${\hat{h}}_{k}(i,\;j)$ denotes the predicted activation at position (i, j). The predicted center of tool *k*, ${\hat{p}}_{k}\left({\hat{x}}_{k},{\hat{y}}_{k}\right)$, is determined as the position (i, j), where ${\hat{h}}_{k}$ attains the maximum value, provided this activation exceeds the threshold µ. Since the model can locate up to two tools, the mean error is employed. The mean error, depending on the Euclidian distance, is computed based on the number of tools (*N*) present in the frame. The Euclidian distance ($d_{kl}$) between the ground truth tool center $p_{l}\;(x_{l},\;y_{l}$) and the predicted center ${\hat{p}}_{k}$ ($\hat{x_{k}},\;\hat{y_{k}})$ is given by (Figure 5a)

(5) $$d_{kl}=\sqrt{{\left(x_{k}-\hat{x_{l}}\right)}^{2}+{\left(y_{k}-\hat{y_{l}}\right)}^{2}},$$

While distance ($d_{kl}$) is commonly measured in pixels [30], it is not suitable for comparison across images of varying sizes/resolutions. Therefore, the distance is normalized relative to the diagonal (diag) of the image,

(6) $$d\;\left(\%\right)=\frac{d\;(px)}{diag\;(px)}\cdot 100.$$

In scenarios where both tools are present (*N* = 2), associating each tool with its respective predicted heatmap ${\hat{h}}_{k}$ is not straightforward, which complicates the calculation of each tool’s center distance. To address this challenge, four distance measurements are computed ($d_{kl}$), representing the distance from each prediction *k* to each tool *l*. Subsequently, the minimum distance among these measures is identified, indicating the model’s optimal prediction for one of the tools. Subsequently, the distance between the other tool and the other predicted point is selected. Expressing the aforementioned conceptually involves considering two real heatmaps $h_{l}$ providing the actual center of each tool $p_{l}\;\left(x_{l},\;y_{l}\right)$, and two predicted heatmaps ${\hat{h}}_{k}$ indicating the predicted center of the tool $p_{k}\;\left(x_{k},\;y_{k}\right)$. The matrix *D* encapsulates the four Euclidean distances $d_{jk}$, calculated as the distance between the real coordinates of each tool $p_{l}$ and the predicted coordinates of each heatmap $p_{k}$ (Figure 5b).

The minimum value of matrix *D* is designated as $d_{1}$, and hence $d_{2}$ is its diagonal,

(7) $$mean\;eror=\frac{d_{1}+d_{2}}{2}\;;\begin{array}{c} d_{1}=min\left(D\right)=d_{kl}\;\\ \;d_{2}=d_{l^{\prime }k^{\prime }}\;/\;l^{\prime }\neq l,\;{\;k}^{\prime }\neq k \end{array};D=\left(\begin{array}{cc} d_{11} & d_{12}\\ d_{21} & d_{22} \end{array}\right).$$

In scenarios where only one tool is present in the image (*N* = 1), the ground truth always corresponds to l=1 since $h_{2}$ is entirely populated with zeros. Consequently, the mean error is computed based on the distance between $p_{1}\;(x_{1},\;y_{1}$) and ${\hat{p}}_{k}$ ($\hat{x_{k}},\;\hat{y_{k}})$, which matches the predicted heatmap with the higher activation, provided it exceeds the threshold *µ*. If neither of the predicted heatmaps exceeds the threshold, the mean error is equal to the diagonal (diag) of the frame, as seen in

(8) $$mean\;error=\left\{\begin{array}{c} d_{11},\;\;\max\left({\hat{h}}_{1}\right)>\max\left({\hat{h}}_{2}\right)\;\&\;\max\left({\hat{h}}_{1}\right)>µ\\ d_{21},\;\;\max\left({\hat{h}}_{1}\right)<\max\left({\hat{h}}_{2}\right)\;\&\;\max\left({\hat{h}}_{2}\right)>µ\\ diag.,\;\max\left({\hat{h}}_{k}\right)<µ\;/\;k\in [1,2]\; \end{array}\right..$$

For images devoid of any tools (*N* = 0), if the maximum activation value of at least one predicted heatmap exceeds the threshold *µ*, the mean error is the diagonal (diag) of the image. Conversely, if the prediction value of both heatmaps is below this threshold, the mean error is zero, as defined by

(9) $$mean\;error=\left\{\begin{array}{c} \;\;\;\;\;\;\;\;\;\;\;\;\;\;\;0,\;\;\max\left({\hat{h}}_{k}\right)<µ/\;k\in [1,2]\\ \mathrm{diag}\left(img\right),\;\;\max\left({\hat{h}}_{k}\right)>µ/\;k\in [1,2] \end{array}\right..$$

2. Accuracy

Accuracy is a measure of the model’s overall performance in correctly classifying instances. It is calculated as the ratio of correct predictions to the total number of predictions. In binary classification, accuracy can be calculated in terms of positive and negative instances as

(10) $$Accuracy=\frac{TP+TN}{TP+TN+FP+FN}$$

**Figure 6:**
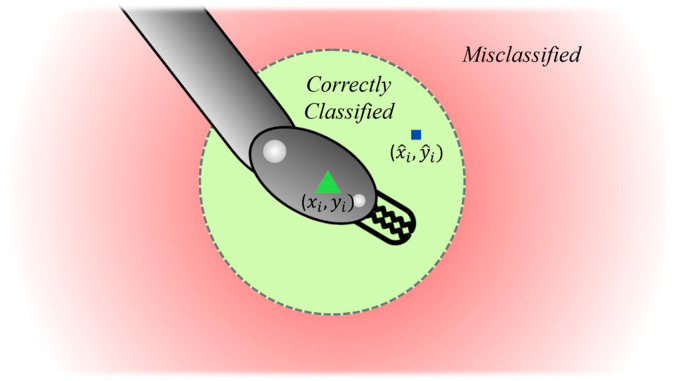
Determination of True Positive (TP) and False Negative (FN) based on tool distance (distance between ground center and predicted center) and a tool distance threshold.

Typically, the Intersection over Union (IoU) ratio is used to distinguish positive and negative instances in object detection tasks. IoU calculates the overlap between the predicted and ground truth bounding boxes [31]. However, in this specific context, the lack of bounding box location and dimension information in some databases makes IoU unusable. Instead, tool distance is employed as an alternative (Figure 6).

A distance threshold (*ε*) is established to discern positive and negative instances, and it can be adjusted based on the specific requirements. In this case, a threshold of 10% of the image width is implemented to rigorously evaluate the algorithm; like previous approaches that often define the threshold in pixel terms [30], this study establishes the threshold as a percentage relative to the image width. This approach enables comparisons across different image sizes and facilitates direct comparisons with models of similar output sizes.

3. Peak Activation Value (PAV)

The peak activation value (PAV) serves as a measure of the confidence of the model in its predictions. It represents the highest activation value within the predicted heatmaps, indicating the network’s highest confidence level regarding the predicted tool location. A PAV close to 1 indicates a high confidence level, while closer to 0 implies lower confidence. The calculation of the PAV depends on the number of tools present in the frame (*N*), as defined by

(11) $$PAV=\left\{\begin{array}{c} \frac{\max\left({\hat{h}}_{1}\right)+\max\left({\hat{h}}_{2}\right)}{2}\;\;\;\;\;\;\;\;\;\;\;\;\;\;\;\;\;\;\;\;\;\;\;\;\;\;\;\;\;\;\;\;\;\;\;\;if\;N=2\\ \max\left(\;{\hat{h}}_{1},\;{\hat{h}}_{2}\right)\;\;\;\;\;\;\;\;\;\;\;\;\;\;\;\;\;\;\;\;\;\;\;\;\;\;\;\;\;\;\;\;\;\;\;\;\;\;if\;N=1.\\ 1-\max\left(\;{\hat{h}}_{1},\;{\hat{h}}_{2}\right)\;\;\;\;\;\;\;\;\;\;\;\;\;\;\;\;\;\;\;\;\;\;\;\;\;\;\;\;\;\;\;if\;N=0 \end{array}\right.$$

For two tools, the PAV is the mean of the maximum activation values of the two heatmaps. For one tool, the PAV corresponds to the maximum activation value within the two heatmaps. In the absence of any tools, the PAV is calculated as one minus the maximum activation value within the two heatmaps.

### 2.4. Model Explicability Using Grad-CAM

Understanding machine learning models is crucial, as it allows us to interpret their decisions and gain insights into their functioning. Treating these models as black boxes can lead to limitations in their applicability and reliability. Comprehending the inner workings of the models allows biases, limitations, and potential areas for improvement to be identified. Therefore, striving for transparency and interpretability is essential for fostering trust, improving performance, and promoting ethical AI practices.

The Grad-CAM (Gradient-weighted Class Activation Mapping) method [32] is a technique designed to provide visual explanations for decisions made by a wide range of CNN-based models, thereby enhancing transparency. This method generates heatmaps that illustrate which regions of the input image were deemed most significant by the model. Such analyses offer insights not only into the rationale behind the model’s decisions but also into the identification of artifacts that may impair prediction accuracy. Consequently, this information can be leveraged to refine the model and improve its performance.

Grad-CAM is a method that uses the gradients of any target concept (in this study, the value of the pixel marked as the center of the tool) flowing into the final convolutional layer to produce a coarse localization map where it is possible to see the important regions in the image that were useful for predicting the concept. This method is applicable to many types of CNN models.

In accordance with [32], to generate the class-specific localization map Grad-CAM $L_{\mathrm{Grad-CAM}}^{c}$, it is essential to calculate the gradient of the score for class *c* in one or both heatmaps (${\hat{h}}^{c}$) with respect to feature maps $A^{k}$ of the convolutional layer intended for visualization $\frac{{\partial \hat{h}}^{c}}{\partial A_{ij}^{k}}$. In our implementation, each class corresponds to each pixel, as it represents a potential location for the tool’s center point. The score in each pixel of $\hat{h}$ represents the probability that the point belongs to the center of the tool. In this case, the class *c* corresponds to the pixel where the center of the tool is really located, and ${\hat{h}}^{c}$ corresponds to its score. The neuron importance weights $\alpha_{k}^{c}$ are deduced by calculating the global-average-pooled gradients flowing back, resulting in

(12) $$\alpha_{k}^{c}=\frac{1}{Z}\sum_{i}\sum_{j}\frac{{\partial \hat{h}}^{c}}{\partial A_{ij}^{k}}.$$

with the weight $\alpha_{k}^{c}$, which captures the importance of a feature map *k* for the target pixel *c*. The final $L_{\mathrm{Grad-CAM}}^{c}$ is obtained by performing a weighted combination of forward activating maps followed by a ReLU, as

(13) $$L_{\mathrm{Grad-CAM}}^{c}=ReLU\sum_{k}\alpha_{k}^{c}A^{k}.$$

## 3. Results

### 3.1. Model Performance

Three models were proposed (Table 2). The first one (H1) has an input size of 512 × 512 pixels and a maximum number of filters in its deepest layer of 256. The second one (H2) has the same input size of 512 × 512 pixels, but only 128 filters in its deepest layer, aiming to reduce the number of parameters of the model. Finally, the third model (H3) has 128 filters in its deepest layer, but in this case, the input size is 256 × 256 pixels with the intention of speeding up the model’s inference by reducing the computational load on the image. The decrease in the number of filters results in a reduction in parameters from 1,946,068 to 490,228. Regarding model H3, the reduction in input size leads to an additional reduction in parameters from 490,228 to 443,854.

Models H1 to H3 underwent training and evaluation using the ATLAS Dione dataset. The dataset was split into a 70:20:10 ratio for training, testing, and validation, respectively. Models were evaluated in a device equipped with an Intel Core i7-7700 processor and 16 GB of RAM. The performance of models H1-H3 is shown in Table 3, in which mean error, accuracy, PAV, and FPS are detailed. Model H1, the largest model, achieved the best results with an accuracy of 89.25% and a mean error of 2.03%. Notably, despite the significant reduction in parameters from model H1 to H3 by almost four times, model H3 exhibited slightly lower performance than H1, with an accuracy of 88.36% and a mean error of 2.66%. This reduction in parameters resulted in a notable improvement in processing time. Specifically, the processing rate doubled from 10.89 FPS for H1 to 27.64 FPS for H3.

Based on the outcomes, a new iteration, H3*, was conducted. H3* was trained on the ATLAS Dione dataset alongside the ITAP Medical Robotics dataset to broaden its exposure to diverse surgical tools and environments. Despite being the same model, H3* showed improved performance over H3, attributed to the increased variability in the training images. The architecture design of the H3* model is visually depicted in Figure S1.

**Figure 7:**
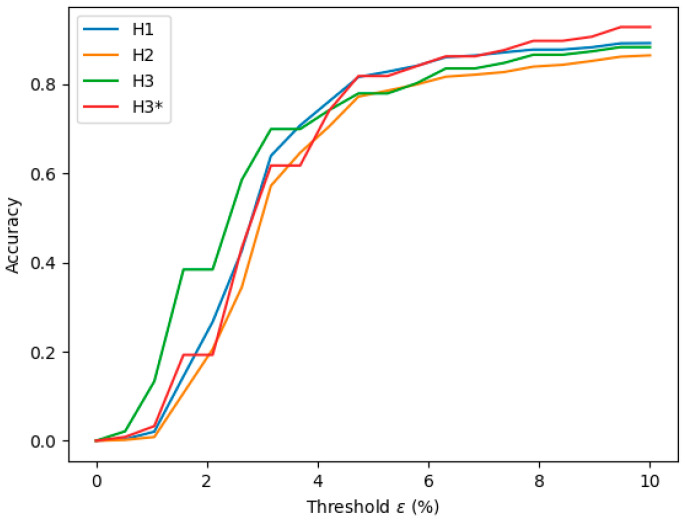
Accuracy comparison between the models as a function of the threshold **ε**.

The models’ performance was also evaluated by varying the accuracy threshold *ε* (Figure 7). Model H3 outperformed all others for error thresholds below 3%, whereas for thresholds exceeding 3%, H3* exhibited superior performance. Model H2 demonstrated the poorest performance across all analyzed error threshold ranges.

Ultimately, the models underwent evaluation using the EndoVis dataset, which consists of only 180 images, insufficient for comprehensive training. Hence, this dataset was used to further evaluate the models’ performance on markedly dissimilar data from those used during training. As shown in Table 4, the model performance decreased on this dataset, given the varying tool types, image backgrounds, and lens focuses. The model that achieved the best generalization performance was H3*, which was the simplest one but was trained with two datasets (ATLAS Dione and ITAP). This result was anticipated given its exposure to a broader range of image variations compared to models H1 and H2, which were trained solely with the ATLAS Dione dataset.

However, the disparity in performance between models H1 and H2 was somewhat unexpected. While model H1, featuring 256 filters, exhibited superior accuracy over model H2, with 128 filters, on the ATLAS Dione dataset (89.25% compared to 86.50%), its performance diminished when evaluated on the EndoVis dataset. Model H1 achieved inferior accuracy compared to model H2 on this dataset (31.52% compared to 35.33%, respectively). This observation suggests a potential overfitting scenario, where the more complex model (H1) may have excessively adapted to the specific features of the ATLAS Dione dataset due to its increased number of filters. Consequently, while the models performed well on images resembling those in its training set, they struggled to generalize effectively to datasets with diverse tool types and background structures, leading to diminished performance in such scenarios.

### 3.2. Model Analysis

**Figure 8:**
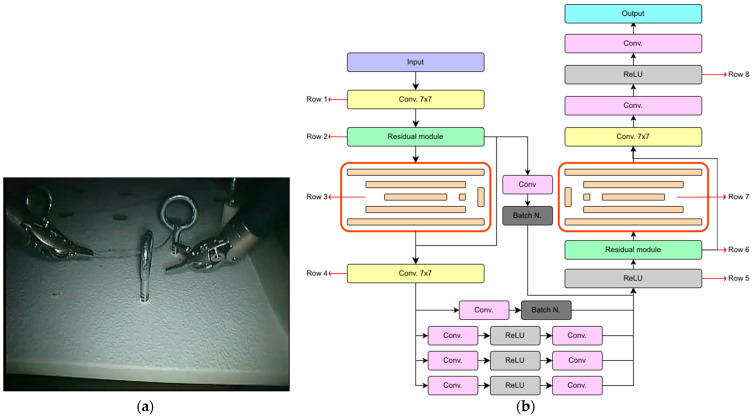
Model analysis with Grad-CAM: (**a**) input image; (**b**) model overview with analyzed portions highlighted.

**Figure 9:**
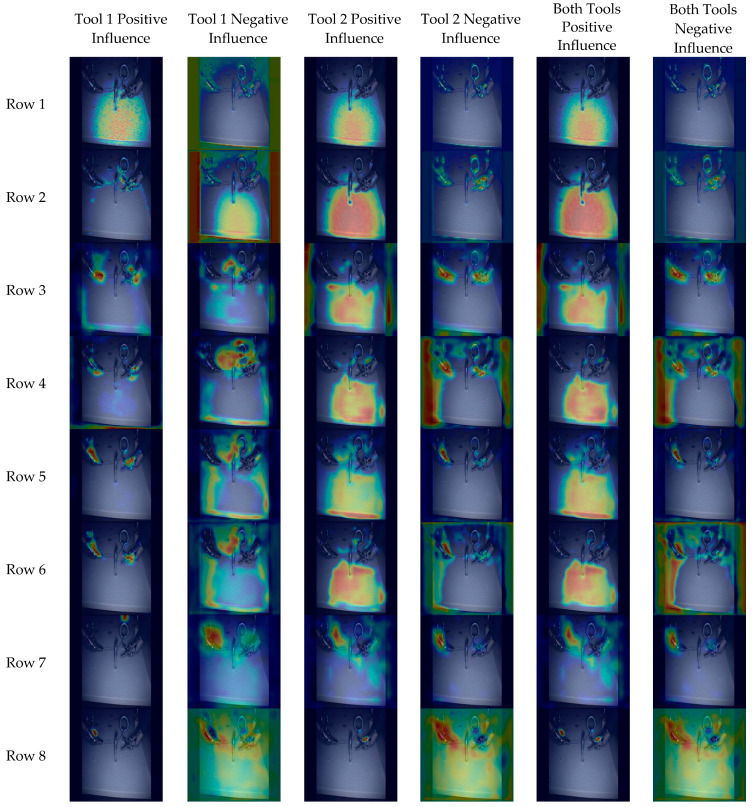
Grad-CAM attention heatmaps for the eight analyzed layers for tool 1, tool 2, and both tools.

This section aims to analyze the proposed model H3* to elucidate the areas where the model focuses its attention on predicting tool centers. For this analysis, the Grad-CAM method was employed, and Figure 8a was chosen due to its diverse object composition and varying luminosity levels, presenting a challenging prediction scenario. The examination encompasses eight distinct segments of the model (highlighted in red in Figure 8b), providing insight into the attention heatmap across the primary modules of the network. Furthermore, attention was directed not only towards the heatmap for the final output but also towards the heatmaps for the output of each tool separately, with the objective of discerning potential disparities in the significance of regions for each tool. The results of the model analysis for each layer are depicted in Figure 9.

In the first layer (Figure 9, row 1), the model focuses its attention on small-size features such as small brightness or texture features in the background. After the residual module (Figure 9, row 2), the model discerns increasingly intricate features, particularly the edges of tools. Additionally, it distinguishes the background, delineating the contours of the tools from the surrounding image space and avoiding the detection of the tool center within these regions. In contrast, the model pays attention to details belonging to other structures, such as metal rings.

**Figure 10:**
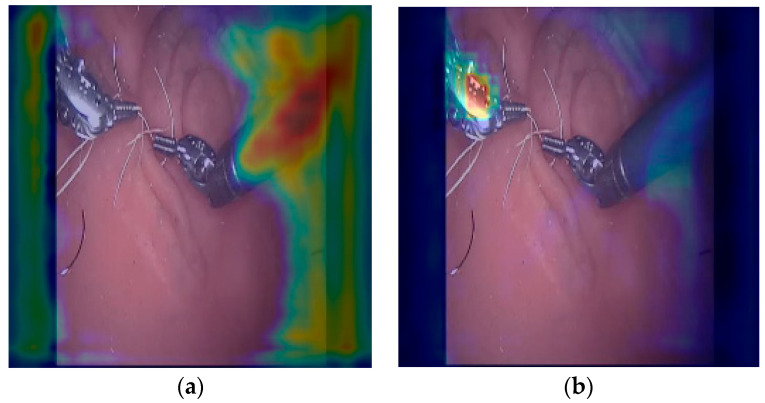
(**a**) Attention heatmap at the end of the first Hourglass module, where the tool’s body is enhanced. (**b**) Negative influence attention heatmap for the right tool at the end of the second Hourglass module, where the left tool is enhanced.

At the output of the first Hourglass module (Figure 9, row 4), it becomes evident that the model has effectively extracted numerous features across various scales, but without complete integration. Therefore, a subsequent module is necessary to undertake this task. As depicted in Figure 9 (row 5), the features extracted by the Hourglass module are integrated to facilitate center predictions. However, it is noteworthy that, at this stage, the tool has yet to assume a pivotal role in the model’s architecture. Notably, for tool 1, the model focuses not only on the image background but also on the presence of another tool, and in other instances, on the body of the surgical tool. This is due to the model’s task of predicting the center of the distal part of the tool, where the tool’s body could potentially act as an artifact in this process. In the case of the other tool, it is equally critical to distinguish between the tool and the background, as well as to differentiate between one tool and another (as illustrated in Figure 10).

The second Hourglass module (row 8, Figure 9) enables more precise localization of tool centers compared to its predecessor, which primarily focused on discerning tool shapes and other image components. In predicting the tool center, the model considers its spatial placement relative to the background and the other tool. Consequently, the significance of the negative influence heatmaps lies in their provision of information regarding these elements to which the model assigns greater importance.

## 4. Discussion

The results of an analysis of the performance of models trained and tested on the ATLAS Dione dataset (models H1–H3) (Table 3) indicated that model H1, characterized by a higher number of filters (256) and a larger input size (512 × 512), demonstrated superior performance for an error threshold of 10%. The latest iteration, model H3*, also trained with the ITAP dataset, surpassed all previous models (H1–H3) for error thresholds exceeding 3%. This improvement is attributed to its exposure to a more diverse training dataset, closely resembling real-world scenarios. The H3* model meets the soft real-time demands for robotic surgery with a processing capability of 27.64 FPS on a standard computer equipped with an Intel Core i7-7700 CPU and 16 GB of RAM. While the model’s speed can benefit from more powerful hardware, such enhancements are unnecessary for these applications.

The models’ performance when tested on the EndoVis Challenge dataset notably lagged behind their performance on the training set, with accuracy dropping from 86–92% to 31–42%. This discrepancy could be attributed to the disparity between the test set (EndoVis Challenge dataset), composed of highly realistic images from ex vivo surgeries, and the training set (ATLAS Dione dataset), which features considerably less realistic images (Figure 2). The choice to train the model on the ATLAS Dione dataset was driven by its extensive collection of labeled images (22,467), in stark contrast to the limited number of labeled images in the EndoVis Challenge Dataset (only 180) (Table 1).

Moreover, a notable disparity exists in the labeling conventions between the datasets utilized. While the ITAP and ATLAS Dione datasets employ a bounding box to encapsulate the tool, with the center corresponding to the center of the distal part of the surgical instrument, the EndoVis Challenge Dataset defines the tool’s center as the boundary between the rigid part of the surgical instrument and the tool, located in its most distal region. Consequently, this discrepancy in labeling conventions introduces further complexity and potential ambiguity in model training and evaluation. This can be observed in detail through the Grad-CAM analysis, as shown in Figure S2. Future directions should consider refining the localization of heatmap centers to better align with tool centers, potentially through methods like center of mass calculation from segmentation masks, to enhance applicability in surgical robotics applications.

Moreover, the proposed algorithm can concurrently detect up to two rigid-link surgical instruments, as available databases feature such scenarios. While the algorithm is theoretically capable of detecting more than two tools with appropriate image training, rigorous evaluation and testing would be necessary. Similarly, the model is also expected to perform well in detecting other surgical tools based on monolithic compliant structures [33,34], as suggested by the Grad-CAM analysis (Figure 9 and Figure 10a), which indicates that the model also relies on the tool’s shape to determine the tip’s heatmap. However, the generalizability of the model is limited, posing a significant challenge. This limitation may not be a concern if the model is trained on a dataset more closely resembling the images encountered in the specific application of robotic surgery for which it is intended. This underscores the need for representative training data to ensure optimal models in real-world scenarios.

Employing Grad-CAM to analyze the model has revealed its proficiency in identifying the primary features of laparoscopic tools and localizing their coordinates by distinguishing them from the background. However, the model also exhibits attention towards features such as reflections, which occasionally lead to misidentifications with other metallic objects. This is evident in Figure 10, where attention is drawn to one of the two metallic rings. The reflective surfaces commonly found on surgical tools are pivotal for the model, given the initial layer’s emphasis on these features. Nonetheless, the presence of other metallic objects or tissue reflections encountered during laparoscopic procedures could lead to confusion. To address this, augmenting the model training with a higher frequency of images containing such objects or tissues could enhance its performance in such scenarios.

The primary objective lies in accurately localizing surgical tools to facilitate real-time robotic endoscope guidance during surgical procedures. While high accuracy is crucial, the model’s ability to provide real-time data to the robotic system is also essential, making a trade-off between accuracy and processing speed necessary. Operating at an approximate frame rate of 30 FPS, the model demonstrates suitability for surgical applications. Additionally, the model underwent rigorous accuracy assessment, utilizing a stringent 10% threshold (*ε*) to ensure the tool localization would be at the center of the field of view (FoV). However, in practical surgical scenarios, relaxing *ε* to 30% still guarantees tool visibility without compromising procedural efficacy. While this paper emphasizes stringent evaluation criteria, future research should delve into assessing the model’s efficacy in maintaining tools within the FoV during surgical operations, necessitating real-time adjustments of the endoscope by the robotic system based on model outputs. For instance, the model’s outputs could be filtered to smooth and eliminate extreme points, enhancing stability despite random noise and occasional misidentifications (e.g., reflections or metallic objects). Another scenario involves the surgical tool being occluded (e.g., by tissue, blood, or smoke), causing the model to output no detected tools. Consequently, the robot would halt until the tool reappears within the FoV and then relocate accordingly. Given the model’s performance, it appears feasible to utilize it for robotic control, and this potential warrants further investigation.

## 5. Conclusions

A vision model has been developed for the localization of up to two tools. This vision model is based on two serial Hourglass modules, which output two heatmaps where the maximum activation indicates the tool center. The model has demonstrated high accuracy and a high frame rate, making it suitable for integration into robotic systems to move the endoscope in laparoscopic surgeries. This makes it possible to partially automate surgery, eliminating the need for an assistant or the surgeon to manually manipulate the endoscope to maintain the operative area in the field of view. While the results of this model are promising, it must be integrated into a robotic system, and its performance must be validated in the specific application, namely laparoscopic surgery.

## Figures and Tables

**Table 1:** Overview of the three datasets utilized in this work: ITAP Medical Robotics, ATLAS Dione, and EndoVis Challenge.

Data	ITAP Medical Robotics	ATLAS Dione	EndoVis Challenge
Frames	3532	22,467	4535
Labeled frames	3532 (100%)	22,467 (100%)	180 (3.9%)
Frames labeled with tool presence	609 (17.24%)	22,467 (100%)	180 (3.9%)
Frame size (px)	720 × 576	854 × 480	640 × 480
Realistic Images	Yes	No	Yes
Label type	Bounding box	Bounding box	Tool center coordinates

**Table 2:** Characteristics of the three proposed models based on the Hourglass network, including number of filters, input size, and number of parameters.

Model	Number of Filters	Input Size (px)	Number of Parameters
H1	256	512 × 512	1,946,068
H2	128	512 × 512	490,228
H3	128	256 × 256	443,854

**Table 3:** Performance evaluation of the proposed models.

Model	N Filters	Input Size (px)	Mean Error (%) (µ = 10%)	Accuracy (%) (*ε* = 10%)	PAV	FPS
H1	256	512 × 512	2.03	89.25	0.778	10.89
H2	128	512 × 512	3.12	86.50	0.611	15.28
H3	128	256 × 256	2.66	88.36	0.714	27.64
H3*	128	256 × 256	1.72	92.86	0.794	27.64

**Table 4:** Performance of the models validated using the EndoVis dataset.

Model	Mean Error (%) (µ = 10%)	Accuracy (%) (*ε* = 10%)	PAV
H1	39.45	31.52	0.2762
H2	30.27	35.33	0.2811
H3	15.23	36.96	0.2110
H3*	20.70	42.93	0.3034

## Data Availability

The ITAP Medical Robotics Group dataset presented in this study is available on request from the corresponding author. The ATLAS Dione Dataset is available at request from its authors at https://www.roswellpark.org/education/professional-training/atlas-program/research-development/dione-dataset (accessed on 26 June 2024). The Endovis Challenge (Instrument Subchallenge) Dataset is openly available at https://opencas.webarchiv.kit.edu/?q=node/30 (accessed on 26 June 2024).

## Supplementary Materials

The following supporting information can be downloaded at https://github.com/itap-robotica-medica/Tool-Localization- (accessed on 26 June 2024): Figure S1: Architectural design of the H3* model; Figure S2: Grad-CAM attention heatmaps for the eight analyzed layers for tool 1, tool 2, and both tools; code is also available at https://github.com/itap-robotica-medica/Tool-Localization- (accessed on 26 June 2024).
